# Stunting and associated factors in children of less than five years: A hospital-based study

**DOI:** 10.12669/pjms.36.3.1370

**Published:** 2020

**Authors:** Sehrish Fatima, Iram Manzoor, Aneeqa Mumtaz Joya, Shehzeen Arif, Sadia Qayyum

**Affiliations:** 1 Sehrish Fatima, Students of 4^th^ Year MBBS, Community Medicine, Akhtar Saeed Medical and Dental College, Lahore, Pakistan; 2 Prof. Dr. Iram Manzoor, MBBS, FCPS, MSC, MCPS-HPE. Director Medical Education, HOD of Community Medicine, Akhtar Saeed Medical and Dental College, Lahore, Pakistan; 3 Dr. Aneeqa Mumtaz Joya, MBBS, MCPS, MPH. Assistant Professor, Department of Community Medicine, Akhtar Saeed Medical and Dental College, Lahore, Pakistan; 4 Shehzeen Arif, Students of 4^th^ Year MBBS, Community Medicine, Akhtar Saeed Medical and Dental College, Lahore, Pakistan; 5 Sadia Qayyum, Students of 4^th^ Year MBBS, Community Medicine, Akhtar Saeed Medical and Dental College, Lahore, Pakistan

**Keywords:** Stunting, Vaccination status, Risk factors

## Abstract

**Objectives:**

To estimate frequency of stunting and associated factors in children aged less than five years in a tertiary care hospital of Lahore.

**Methods:**

An Analytical cross-sectional study was conducted in Pediatrics Outpatient Department at Akhtar Saeed Trust Teaching Hospital, Lahore from December 2017 to July 2018. Two hundred children of ages under five years coming to outdoor for treatment of minor ailments were included after informed consent from their parents. Non-probability, convenient sampling technique was used to collect sample. Data collected and analyzed on SPSS version 19. To find out association of stunting with multiple qualitative variables, chi-square test was applied and p-value was fixed at ≤ 0.05 to be significant.

**Results:**

Out of 200 children screened in OPD, 42 (21.0%) were found to be stunted. The total percentage of stunting in male children was 28 (66.6%) and in female children were 14 (33.3%). Stunting was significantly associate with male gender (p=0.047), joint family system (p=0.049), low literacy level in mothers (p=0.031), unvaccinated status (p=0.003) and history of bottle feeding (p=0.037).

**Conclusion:**

The frequency of stunting in children less than five years of age is 42 (21.0%). The significant risk factors associated with stunting were found as male gender (p= 0.047), joint family system (p=0.049), low maternal education (p=0.031), unvaccinated status(p=0.03).

## INTRODUCTION

Stunting is termed as reduced height for age in a sample population.^1^ It is a major public health issue worldwide.^2^ Stunted growths is one of the contributory factors of high mortality among children in low and middle income countries.^3^

The ideal development of children is a basic human right and an aim of health systems and social security.^4^ Internationally, it is estimated that 22.9% children under the age of five have growth hindrance. From 2000 to 2016, stunting gradually declined from 33.7% to 22.9% and the numbers tumbled from 198 million to 155 million.^5^ In 2017, a survey was conducted in thirty-six high burden countries and results showed that one out of two stunted children lived in Asia and one out of three in Africa^6^ In developing countries, 200 million children under age five years are influenced by retarded linear growth and in those 40% fall in the category of iron deficiency anemia.^7^

Pakistan is one of the ten nations in the world where a greater part of less than five year old population is affected by stunting.^8^ As per the current situation in Pakistan, it is evaluated that almost 44% of children are stunted with predominance being marginally higher in males (48%) than in females (42%).^8^ In total, with 12 million stunted children Pakistan is positioned the third in the world.^9^

The major risk factor for direct cause of stunting includes poor maternal health, lack of antenatal care facilities, insufficient feeding and care, insufficient infrastructure and healthcare facilities.^10^ The economic wealth is a second major fundamental issue for dietary change.^11^ Maternal and paternal education also plays a vital role in undernutrition leading to a greater chance of stunting during primary school students.^12^ Relationship between younger ages of mother, birth spacing and intention of the couple for expecting a child, each has a huge association to hindered growth.^13^ Poor sanitation is a noteworthy general wellbeing concern connected to a few results of stunting.^14^ Absence of sanitation and open defecation contributes to diarrhea and spread of intestinal parasites which in return cause lack of healthy sustenance.^15^ Stunting in early life is associated with unfriendly consequences including poor development and instructive performance, low wages and lost productivity.^16^ The objective of this study was to assess the frequency and its relationship with sociodemographic profile and feeding practices which cause stunting among children.

## METHODS

A cross sectional survey was conducted at an outpatient department of pediatrics of Akhtar Saeed Trust Hospital during December 2017 to July 2018. This study was conducted on a sample of 200 children, less than five years coming to outdoor for treatment of minor ailments. Data was collected after taking consent from the parents. Children with congenital and severe illness were excluded. Before data collection of this study, IRB approval (No. M-18/21/-CM) was taken in February 6, 2018. Non probability convenient sampling technique was used to recruit sample of 200 children. A structured questionnaire was used to collect data regarding sociodemographic profile, height and feeding practices of these children. Data was entered and analyzed in SPSS version 19 and was presented in the form of frequency tables and graphs. Chi square test of significance was applied to find out association between sociodemographic factors and stunting pattern and p value was fixed at ≤ 0.05 to declare results as significant.

## RESULTS

**Fig.1:**
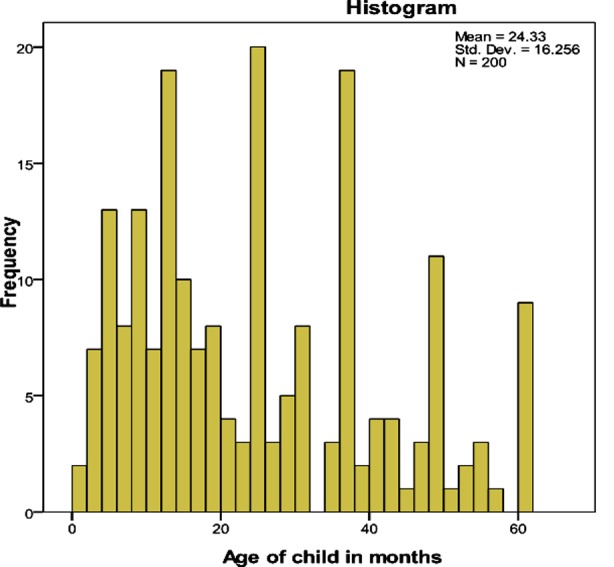
Graphical distribution of age in children (in months).

The mean age of the sample was 24.33 ±16.256 months (Fig.1).

There were 200 participants in total, out of which 110 (55%) males and 90 (45%) females respectively. One hundred and sixteen (58%) participants belonged to urban areas. Out of 200, 105 (52.5%) belonged to joint family system. Educational status for fathers showed only 32 (16%) had done graduation and post-graduation. Same results were obtained with maternal education status and higher education was observed only in 27 (13.5%). Drinking water supply available in households was through government source in only 106 (53%). The mean income was estimated to be Rs. 20,300. 123±13,759.96 (Table-I).

**Table-I:** Sociodemographic profile of children less than five years of age.

	Frequency(n)	Percentage (%)
*Gender distribution*		
Male	110	55
Female	90	45
*Area of distribution*		
Rural	84	42
Urban	116	58
*Family type*		
Nuclear	95	47.5
Joint	105	52.5
*Educational status of mothers*		
Uneducated	69	34.5
Primary	79	39.5
Secondary	25	12.5
Graduate	20	10.0
Postgraduate	7	3.5
*Educational status of fathers*		
Uneducated	64	32.0
Primary	67	33.5
Secondary	37	18.5
Graduate	18	9.0
Postgraduate	14	7.0
*Monthly income*		
Below Rs 20,000	123	61.5
Above Rs 20,000	77	38.5

One hundred sixteen (58%) children had complete vaccination status, 79 (39.5%) for incomplete and 5 (2.5%) never received even a single dose of vaccination.

History of breastfeeding was found in 166(83%) of the children but it was also accompanied with bottle feeding in 161(80.5%). Only 19.5% of the participants were exclusively breastfed by their mothers. About 22.5% of the mothers believed that their feeding practices are inadequate for their kids (Table-II) Majority of the participants relied on three meals per day. Mean meals 2.99±1.109 were taken.

**Table-II:** Feeding pattern of children.

	Frequency (n)	Percentage (%)
*Feeding practices*		
4 or more small meals (Adequate feeding)	155	77.5
Less than 4 small meals (Inadequate feeding)	45	22.5
*Breast feeding*		
Yes	166	83
No	34	17
*Bottle feeding*		
Yes	161	80.5
No	39	19.5

**Fig.2:**
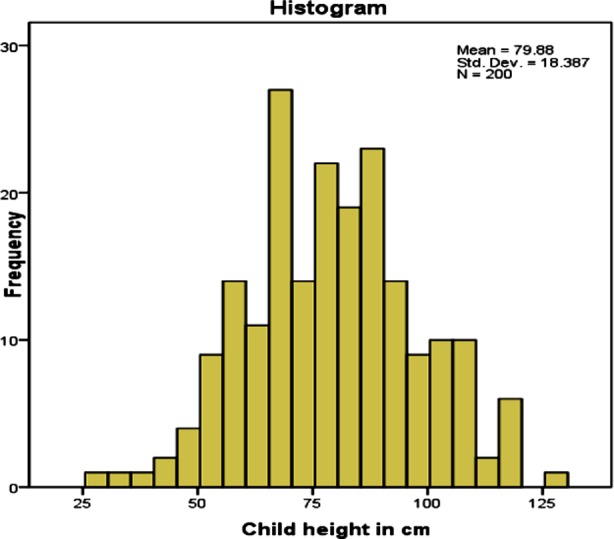
Mean distribution according to height of participants.

The mean height was 78.88 ± 19.258 cm (Fig.2). The researchers have used reference curves developed by WHO to assess the status of stunting. After application of test of significance, results showed that stunting was strongly associated with male gender (p=0.047), joint family system (p=0.049), maternal education (0.031), incomplete vaccination status (0.003) and history of bottle feeding (0.037).

## DISCUSSION

The study findings showed significant association with male gender of the child which is considered as being a solid determinant of stunting in children under five years of age. Some past studies from Sub Saharan Africa have shown that males are increasingly affected when contrasted with females.^17^ There is a common conviction that in Pakistan parents are strict with their daughters than their sons and often give unique consideration to the male child is not supported by this survey.

Consistent with findings of other similar studies there was no significant urban-rural disparity with stunted growth in this study. Patients who reported to the hospital resided in nearby vicinity and had availability to better healthcare facilities, water supply and improved sanitation. A study done in Bangladesh suggests that more than fifty percent living in urban slums had a higher prevalence of stunting.^18^ Contrary to this, another study conducted in Gaza in 2010 suggests predominance of stunting in the urban territory was higher than the rural areas.^19^ This outcome could be due to poor economic conditions.

The results of this study showed significant association between stunted growth and joint family system. A study conducted in a village of Nepal suggested that children living in joint families were found to be less inclined to being underweight and stunted. This was due to the fact that mothers are field workers and they might be physically unfit to nurture their newborn consistently. In such circumstances, in joint families, there are dependable people like grandparents.^20^ But these findings don’t match with results of this study due to high dependency ratio in joint family systems of Pakistan.

Maternal education has been demonstrated to be significant for a child’s wellbeing and survival. Similar findings were observed in this study showing, significant link between stunted growth and lower maternal education status. According to a study carried out in Bolivia there was significant association between them.^21^ There is proof from Indonesia and Bangladesh that maternal education has a protective impact on stunting.^22^ Contrary to these findings there was no significant association found between educational status of fathers and stunting.

The first thousand days of life are a critical period to prevent risk factors of stunting.^16^ Therefore; more consideration ought to be given to the wellbeing and living conditions occurring at that developmental stage. Analysis of this study showed significant association between bottle feeding practices and stunted growth of children. Some findings have been supported by other developing countries, as a study on Malawian infants suggested that administration of any other fluid other than breast milk particularly before the age of four months, is related with gastrointestinal issues. This may result in stunting, micronutrient insufficiencies and vulnerability towards different illness within the first few years of life.^23^ In our study, food insecurity was excluded as a related factor of stunting, but various studies have reported that it influences the wellbeing and has been linked to child’s intake and weight.^24^,^25^ Food insecurity might be identified with protein energy malnutrition, which was obvious in cases of stunting.^26^,^27^

The significant result in our survey was that the incomplete/no vaccination status were strongly associated with stunting. A study carried out in Nairobi had similar findings.^28^ In Tanzania, Zambia, Madagascar and Zimbabwe immunization were given altogether and as a result dietary status was improved and health gains were achieved.^29^ Immunization in children is a protective measure in order to avoid various childhood diseases and stunting.^30^ To reduce the burden of stunting in developing countries, major emphasis should be laid on improving the sociodemographic profile, promotion of breast feeding and vaccination practices.

### Limitations of the study

Data based on a hospital-based sample. Such studies should be conducted in communities to have comparable results.

## CONCLUSION

Male gender, children residing in joint family system, children with uneducated mothers, children with incomplete/no vaccination history and fed on bottle feeding are at high risk of developing stunting.

### Recommendations

Promotion of exclusive breast feeding and vaccination practices among parents can reduce burden of stunting in Pakistani children.

### Authors’ Contribution:

**SF:** Conceptualization, discussion write up.

**IM:** Analysis and results write up. Accountable for accuracy and integrity.

**AMJ:** Introduction write up.

**SA:** Questionnaire development, data collection.

**SQ:** Methodology write up and data collection.
